# Nursing staff’s experience of appearance issues in various nursing situations

**DOI:** 10.1186/s12912-021-00731-y

**Published:** 2021-10-20

**Authors:** Åsa Bringsén, Johanna Sjöbeck, Pia Petersson

**Affiliations:** Faculty of Health Science, Kristianstad University, Kristianstad, Sweden

**Keywords:** Appearance, Nursing, Health, Empowerment, Person-centred care

## Abstract

**Background:**

Health care professionals frequently interact with unknown patients in a process involving appearance-based judgements and priority-setting, all of which has an effect on health care equality. The healthcare provider–patient interaction is also highly relevant for the awareness and support of patients’ appearance concerns, with an associated possibility for improving patients’ satisfaction with their appearance and health. The aim was therefore to explore nursing staff’s experience of patients’ appearance issues in various nursing situations, with the purpose to facilitate awareness raising and knowledge development.

**Method:**

A qualitative research approach with focus group interviews was chosen due to the exploratory aim of the study. Five semi-structured focus group interviews were conducted with 24 nursing staff in total (19 women and five men). The participants’ ages varied (20 to 45 years) as did their professional nursing experience. The interviews lasted approximately one hour, were digitally recorded, transcribed verbatim and analysed through thematic analysis.

**Results:**

The thematic analysis resulted in the two themes *Patient perspective* and *Professional nursing role*, with associated subthemes. The findings showed the importance and impact of appearance issues in nursing situations and how these are linked to the health of the patients. Some groups of patients were identified as more vulnerable than others, which was associated with health care inequalities and health disparities. Value-based strategies along with knowledge, and skills for holistic person-centred care were identified as important resources for the development of appearance-related awareness and support in various nursing situations.

**Conclusion:**

Strategies for improvement can be realised through the educational system for nursing staff, but mainly by using collective reflective learning forums in different workplaces. An empowerment approach is considered a useful framework for the implementation of holistic person-centred care, functioning as a resource for appearance-related awareness and support in various nursing situations. However, more research is needed on the complex and challenging phenomenon of appearance issues in nursing situations. Knowledge development related to successful person-centred strategies for appearance-related awareness and support is important, especially strategies with a salutogenic perspective.

## Background

Most post-industrial societies are characterised by a so-called appearance culture, where appearance is highly significant and people are continuously being exposed to quite narrow minded messages about appearance and what to do to achieve an ideal appearance [1–3]. Unfortunately, appearance culture is also related to the widespread phenomenon of dissatisfaction with appearance [1, 3–5], which in turn is associated with a variety of negative health effects [1, 6–8]. Research focusing on positive body image confirms the strong link between appearance, well-being and health, thereby highlighting the importance of appearance-related support by health care providers [8, 9]. Healthcare providers meet patients with a wide range of appearance concerns, such as different disfiguring conditions, disabilities, neurological diseases, and ageing and weight/shape issues, and a need for appearance-specific knowledge and skills has been identified [8, 10, 11]. More research is needed concerning factors of relevance to improve health care providers’ opportunities for knowledge and skills development regarding working with patients’ appearance issues in practice [8].

Health care professionals frequently interact with unknown patients in a process involving appearance-based judgements and conclusions. With no or limited experience of or information about someone, appearance is the initial source on which opinions about a person are based. For instance, research has shown that a brief exposure to an unknown face leads to quite specific personality conclusions about that person [12, 13], with an associated effect on social outcomes [14]. People in general tend to relate positive personality traits to well-known appearance typicality’s [15], which also means that people with atypical appearance have a higher risk of being related to less positive personality traits with associated more negative social outcomes from interaction. Stigmatisation is a widespread negative social outcome that is of relevance regarding appearance issues on the interpersonal level described above, with a direct link to negative health consequences for the exposed [16]. The process of appearance-related first impression interaction thus influences the quality of care and the prerequisites for equality and equivalence of care [17, 18]. Promotion of healthcare professionals’ awareness of appearance-related first impression inferences is therefore also needed [19, 20].

The healthcare provider–patient interaction is thus highly relevant for the identification and support of patients’ appearance issues with an associated possibility for patients’ development of satisfaction with appearance and health. Factors of importance regarding appearance issues in healthcare situations are thus also particularly important to explore, given the described effect on the quality of care, including equality and equivalence of care and associated prerequisites for health of the patients. An exploratory study investigating factors of relevance for appearance issues in various nursing situations was therefore conducted with Swedish nursing staff.

## Methods

### Aim

The aim was to explore nursing staff’s experience of patients’ appearance issues in various nursing situations, with the purpose to facilitate awareness raising and knowledge development.

### Study design

A qualitative research approach with focus group interviews was chosen due to the aim of exploring nursing staff’s experience of patients´ appearance issues in various nursing situations. Focus group interviewing made it possible for the moderator to maintain the focus of the interview throughout and facilitated exploration of the participants’ various opinions without the purpose of reaching an agreement [21]. Semi-structured interviews helped the participants express their experiences in their own way [22].

### Participants

Five semi-structured focus group interviews were conducted with the 24 nursing staff in total (19 women and five men), who accepted the invitation to participate. Their ages varied from approximately 20 to 45 years, with an associated variation in length of professional experience. All participants were also nursing students at a university in the south of Sweden. Eleven of the participants were working as registered nurses studying for a specialist degree in nursing, and 13 were assistant nurses or care assistants studying for a registered nurse’s degree. Their professional experience was related to varying nursing settings, which together included experiences of patients representing the entire lifespan. The five focus group interviews were conducted with four to six participants in each.

### Research process and context

The focus group interviews were conducted between February 2016 and February 2017, with the first one functioning as a pilot interview. The interview guide emanated from the aim of the study and focused on appearance issues in nursing situations and resources needed in relation to appearance issues in nursing situations. The participants were for instance asked to reflect on appearance issues in general and then related appearance issues to their professional experiences from various nursing settings and situations. They were also asked to describe what kind of appearance issues they had encountered in their professional role and how they experienced different nursing situations regarding appearance. The participants were also asked to share their thoughts on different aspects of importance for the described experiences as well as resources needed regarding their experience of appearance issues in their professional role.

A collage of pictures was used as a facilitator for the appearance-related conversations during the interviews. The collage consisted of pictures of people from a variety of situations representing deviations regarding appearance, that is varying age, gender, culture, size, socioeconomic group, and visible differences. The pilot interview functioned quite well but the participants had a tendency to sometimes drift away from the nursing perspective of the study. The moderator therefore led the following focus group interviews with stronger emphasis on the professional perspective and the nursing context. Other than that, no alterations were made during the process of interviewing. The focus of the interviews,the collage of pictures and the interview guide were kept throughout the study and the data from the pilot interview was considered useful and therefore also included in the study. All interviews were conducted with co-author JS as a moderator, together with an assistant observer/moderator who took notes during the interviews and was given the opportunity to ask complementary questions at the end of each interview.

Potential participants were informed about the study written and orally at the university. The inclusion criterion was having professional nursing staff experience, and those interested in participation signed up at a focus group interview adjusted to the schedule of their education. Adjustments to their education resulted in participants taking part in focus group interviews together with other students from their own educational program. The information included the aim, motivation and method of the study, voluntary participation, confidentiality, publication plans and contact information of the researcher in charge of the study. The information also included a form for obtaining written informed consent in accordance with the Swedish law of research ethics, SFS 2003:460. The study was thus planned and carried out in accordance with the the Swedish law of research ethics, which is based on the Helsinki Declaration ensuring that the participants took part in the study voluntarily after providing written informed consent. In agreement with the Swedish law of research ethics (SFS 2003:460), no ethical approval was required since the study focused on the participants’ professional experience.

To promote an open climate during the focus group interviews, the interviews started with the researcher repeating the information about the study and emphasising the importance of the participants not sharing each other’s contributions with others afterwards. The risk of others finding out what different participants shared during the interviews was in this way reduced. Complete confidentiality is however not possible to preserve when using focus group interviews. Each interview lasted approximately one hour and was carried out in a conference room at the university. The interviews were digitally recorded, transcribed verbatim and analysed through thematic analysis [23].

### Analysis

The thematic analysis was performed after completing all interviews and was conducted using an inductive approach due to the exploratory character of the study. All researchers participated actively throughout the thematic analysis, which was supported by the six steps; Familiarisation, Coding, Generating themes, Reviewing themes, Defining and naming themes and finally Presentation of findings [23, 24]. Familiarisation was carried out by listening to the digital recordings and reading the transcripts. The researchers then discussed codes and generated themes using a reflexive mind map technique on a white board. The findings of the study were then presented to, member checked and verified through peer debriefing [24, 25]. The peer debriefing was done with nursing students with professional nursing experience participating in appearance-related lectures within the nursing programme at the university in the following semester. No alterations were made based on the peer debriefing. The review process was based on the researchers critically reflecting together on the content of and relationship among codes, subthemes and themes. The mutual reflection then resulted in the final definition, naming and presentation of findings using the themes, with an associated conclusion that inductive thematic saturation had been reached [26].

## Results

The study resulted in a rich body of material in relation to the aim of the study. The thematic analysis led to a framework with the two themes *Patient perspective* and *Professional nursing role*, with associated subthemes.

### Patient perspective

The participants’ experience of appearance issues in various nursing situations was related to the patient perspective through the subthemes named *Patients’ appearance concerns* and *Vulnerability.*

#### Patients’ appearance concerns

The participants’ experiences showed that appearance issues were important for most patients and a need for appearance-related awareness and support in nursing practice was articulated. The participants described how nursing staffs’ awareness was needed for recognition of appearance issues, which was considered a prerequisite for them supporting patients with appearance concerns. The participants’ experience showed how appearance-related awareness and support in nursing could be related to the patient perspective through the health and well-being of the patients. The participants believed that appearance-related awareness and support helped the patients maintain their confidence and identity through a process of disease and treatment, with an associated effect on physical functioning, and appearance.

*Because they kind of feel better about it, I think it’s a little thing, you know. And it’s the same thing I think about these cancer patients, how important their wigs are. It’s like their identity to a large extent. (FG1).*

The participants had experiences of a variety of patients’ appearance concerns. They described, for instance, how patients wanted to look good and feel fresh in different social situations while hospitalised. Some patients were concerned with hair and wigs, body hair, clothes or makeup, while others wanted to take a shower and/or use perfume before interacting with others. The participants also described nursing situations where appearance concerns were the reasons for the patients requesting care. These patients suffered for instance from eating disorders, muscularity-driven excess intake of supplements, facial congenital disorders, skin conditions, or had undergone plastic surgery or gastric bypass surgery or treatment for gender reassignment or destructive self-harming behaviours. The participants experienced some appearance concerns that were difficult to address and treat because of psychological aspects in general but also due to how the problems could be related to high status behaviours or ideals among the patients.

*I think it’s how hard the patient can push the body, even though it’s not good. To be able to push the body so hard that they end up in hospital, I think that’s the thing.*

*That you have good willpower, you have control over your body. That there is some form of strength in it. (FG4).*

Patients’ appearance concerns were also related to other diagnoses, emphasising the importance of appearance-related awareness and support in nursing in general and not just in situations where appearance is more clearly a main issue for the patients. The participants referred to disease-, treatment- and accident-related physical impacts such as scarring, the loss of body parts, weight change, braces and so forth. The participants thought that patients experience these physical changes in different ways due to the psychological dimension of subjective experience, which contributed to the participants finding patients’ appearance issues difficult to address. The findings indicated that psychological aspects of patients’ physical conditions were more difficult to identify and address in nursing situations and therefore they received less/too little attention in nursing settings compared to the physical and more visible aspects.

*It feels like sometimes issues like this will have to wait, because you usually prioritise the (physically) sick, (…*) *this is not an emergency. It will have to wait.*

*I can sometimes feel that it’s pushed away a bit, which is a shame. (FG5).*

#### Vulnerability

The findings showed how some nursing situations and/or patient groups were experienced as more sensitive or vulnerable regarding appearance issues. Patients’ vulnerability was, by the participants, for instance related to appearance in social situations. Patients with low socioeconomic status, exemplified by homeless patients, were one group of patients perceived to be particularly sensitive in social circumstances. The participants also described experiences of how the patients in general were vulnerable due to illness but also because of they had a subordinate role compared to the expert role of nursing staff and especially doctors. Patients’ vulnerability was then related to appearance issues through experiences of nursing situations when the patients’ body and or appearance were in focus.

*You are a little vulnerable in the hospital. You feel that you are at a disadvantage against the doctors, I think. As a patient they may not really dare to say that they for instance would like to have the curtains drawn around the bed, so not everyone can see, such small things. (FG1).*

The participants’ experiences indicated that it was quite common for patients to feel uncomfortable in situations when their body and/or appearance were under scrutiny in nursing situations. Some of the participants said that female patients were more concerned about appearance issues than men, but the findings also showed that men could be more distressed than they appeared. Older patients were, on the other hand, experienced as less concerned about appearance than other patients. However, older women previously occupied in appearance-focused professions were described as more concerned about appearance than older patients in general. Children were experienced to be better at dealing with appearance-related consequences in hospital settings, compared to adults.

*No one can handle illnesses better than children really, who still have such a joy of life and play, perhaps because they are in a context (hospital setting) where there are other children with similar concerns, so it may be different when they come to school, where they become this visible deviant. (FG4).*

However, children were also experienced to be vulnerable regarding other appearance-related circumstances, for instance exemplified by screening for overweight among children within the school health system. The participants described how school health nurse identification of overweight, and preventive actions in collaboration with parents could do more harm than good in the long run and they discussed the initiative from an ethical point of view.

*Problems with overweight must be raised but is it ethical? It can have terrible consequences in adolescence. (FG4).*

Patients with obesity were also experienced to be a vulnerable group of patients, who often showed signs of embarrassment or shame when their weight became challenging in nursing situations.

*A patient came in who weighed yes, over three hundred kg, who needed to go to the toilet and couldn’t get up and we had no aids available. It was so awful, she felt bad, you could see it in her, like I’m a problem, but I really need to go to the toilet. She experienced herself as a problem then. (FG4).*

Various types of equipment were described as resources regarding patients’ appearance related vulnerability in nursing situations. The participants thought that the equipment required when caring for patients with specific needs, for instance patients with overweight, had to be easily accessible to limit the risk of the patients feeling embarrassment and shame in nursing situations. Equipment for protection of patients’ integrity in general was also experienced as an important resource, for instance when caring for patients in dormitory settings.

### Professional nursing role

A central component of the participants’ experience of appearance issues in various nursing situations was the professional nursing role. The findings related to this theme were organised through the subthemes *Interpersonal nursing approach, Priorities* and *Learning for professional competence.*

#### Interpersonal nursing approach

The participating nursing staff continuously referred to how their interpersonal nursing approach had an important role for appearance-related awareness and support in various nursing settings. The participants emphasised the importance of nursing being characterised by a holistic and person-centred approach. The participants described it as focusing on the whole of the person and not just a patient with a specific concern or diagnosis. A holistic person-centred nursing approach was, by the participants, related to the opportunity for person-adjusted appearance-related awareness and support without stigmatisation and judgement based on preconceptions or prejudices. The patients’ right to equal and equivalent care no matter what ailment they had, who they were or what they looked like was related to professionalism and was considered self-evident in nursing practice. The findings showed, however, varying thoughts about professionalism. Some of the participants believed that as soon as they came to work and put on their nursing outfit, they became professional and treated everybody equally, no matter what. Others said that they reacted to appearance as human beings in general and constantly needed to be aware of this to be able to use it as a resource for person-centredness and eliminate or at least minimise any eventual negative effects on equality of care.

*Yes, so you have them automatically (preconceptions), but then you have to make sure to push them back so that the patient does not see them, because when the patients are there, everyone is of equally worth, regardless of whether you are a Muslim, Christian, fat, slim … it should not matter. (FG3).*

Even though appearance issues were considered important, the participants thought that too much focus by the nursing staff on appearance could have a negative impact on the nursing situation. They stressed the importance of care focusing on the patients’ experience of problems, since the subjective experience of the patient did not always correlate with what was initially visible and identified as a problem by the nursing staff. Focusing most on what the patient was seeking care for was therefore considered a way to promote professionalism regarding appearance-related awareness and support.

Communication with an emphasis on nursing staff listening to the patients was identified as a key component of the person-centred nursing approach needed for appearance-related awareness and support in various nursing situations. Communication ought to be characterised by mutual respect and acknowledging the patient’s integrity, with an associated opportunity for the patient to raise appearance issues if needed. The importance of communicative support was emphasised by the participants. Sufficient time for communication was described as especially important, and sometimes communication templates were requested. The participants thought that the arrival call was an initial opportunity for appearance-related awareness with subsequent eventual need for support and care, even though the experienced time allocated was limited.

*I’m also thinking about this arrival call we have with the patients, where we have a huge area for improvement. You could probably include body image and appearance, in a nice way but I do not know how. (FG4).*

The participants reflected on the sensitivity related to communicating appearance issues with patients and described how relational aspects, for instance personal chemistry, could promote, or hinder the communication. The sensitivity of appearance issues was also related to the motivation of a nonverbal communication strategy for showing the patients that their appearance was unimportant to the participants. Communication could also be related to situations where nursing staff consciously or unconsciously showed that some patients or ailments were looked down upon. The participants described situations where staff talked negatively about patients with each other and how it was difficult to totally conceal feelings or values when interacting with patients afterwards.

*Well, I think it’s very much about us; we in our profession must absolutely not judge in any way in advance, it is simply about kindly finding out if different things are problems for the people we meet. (FG2).*

The participants’ experience showed how nursing experience could be a resource for achieving person-centred care but also an impediment. Experience as a resource was related to staff getting better at understanding and adjusting the care to the various needs of different patients. Experience of being a patient was also highlighted as a resource for person-centredness. Experience as an impediment was related to what the participants referred to as “the habitual effect”. The participants thought that experience could gradually lead to a more routine-based nursing approach in general, at the expense of being observant, reflective, and flexible regarding patients’ specific needs. Nursing staff gradually becoming used to quite severe appearance-related conditions, with an associated risk of underestimating the impact of less severe conditions based on the subjective experience of patients, was also experienced by the participants.

*You get so used to it, that it becomes like nothing really for you; you forget how big it can be for a patient, I think. (FG3).*

The participants’ experience showed how nursing situations were affected by general appearance issues as in any other situation of human interaction. Experienced issues of importance were for instance indicators of patients’ age and gender, attractiveness, weight or size aspects, visible cultural artefacts, or signs of socioeconomic position and so forth. The participants described how they tried to adjust intimate nursing situation to patients through choice of staff based on for instance gender, age, culture or values. The motives for this adjustment were to make the patient feel more at ease and less focused on their appearance, and to facilitate person-centredness and equality and equivalence of care. Experiences of the participants indicated that female nursing staff were generally more accepted by patients in intimate nursing situations. This experience was explained by the nursing profession traditionally being a female-dominated profession but also as a response to men being overrepresented as perpetrators of violence against women in society in general.

All in all, adopting a person-centred nursing approach for appearance-related awareness and support was described as a challenging balancing act involving identifying, understanding, and supporting but without feeling sorry for and/or relying too much on experience-based preconceptions about appearance-related issues.

#### Priorities

The participants’ experiences indicated that appearance issues had a low priority within the healthcare sector in general.

*Like this with gastric bypass, there is sometimes a condescending tone towards those people. Sometimes some in healthcare have difficulty to relate to this with appearance and body image,* etc. *Because we do not see it as a disease that we must do something about, they are not as prioritised. (FG5).*

The participants’ experiences indicated that it was quite common that nursing staff were more positive towards appearance-related treatment within the tax funded healthcare system when it had a physical functionality motive and/or the problem was not self-inflicted. Limited resources available within the tax funded health care system was related to the participants’ thoughts about what the resources ought to be used for, and the participants described how they sometimes acted for some appearance ailments to be better handled and prioritised within the system.

*Sometimes you choose which doctor they should go to, I can say that. They are different, they are different as people and different as doctors. Sometimes you can feel that certain ailments are easier to place with some doctors. (FG5).*

The participants’ experiences showed that care settings could mean various opportunities for appearance-related awareness and support based on the orientation of the setting. The more physical- and functionality-focused orientation of the care setting, the less attention to psychological appearance issues there was, from the participants’ point of view. Participants also perceived a change in primary health care due to their experience of an increase in appearance-related concerns, such as acne and other skin conditions, among patients. Hospital settings were considered equipped with extra resources for prioritisation of appearance issues through the availability of other professions, for instance physiotherapists, occupational therapists, dietitians, curators, and priests. Relatives were also described as a possible resource for appearance-related recognition and support in various nursing situations.

#### Learning for professional competence

A need for learning for professional competence among nursing staff was identified through the experience of the participants in the study. The participants highlighted that there was a need for knowledge about appearance-related issues and communicative skills, and for improvement of appearance-related awareness and support by nursing staff in various situations. The need for knowledge about the psychological dimension of appearance issues in general was emphasised but also the difficulty in understanding and caring for patients from different generations.

Learning within the education system was described as important but more often the importance of collective, reflective learning forums was articulated. A collective, reflective learning forum was described as a systematic, collective learning process based on communication and reflection within workplaces.

*I think you should have it (a reflection forum) at least once a week, best after every work shift, but at least once a week, reflections on what was good, what we did, why did you notice what I didn’t notice, like that. (FG2).*

Experience-based interprofessional reflection where all staff, regardless of profession or type of employment, were included and valued, was requested by the participants. Their experience was however that existing reflection forums often excluded temporary staff and students in practical placement education, and that the hierarchy of different professional roles could be an obstacle.

*But the mentality of the workplace, when we had ethical reflections, the mentality was that I’m just a damn care assistant, (…) I know nothing, it was a bit like that and I felt that I would not say too much, sort of. (FG4).*

Possibilities for improvement of experience-based interprofessional reflection, which could function as a resource for appearance-related awareness and support in various nursing situations, were thus identified.

## Discussion

### Patients’ appearance issues and health

Appearance issues affect nursing situations overall and are therefore considered important for nursing practice in general. The participants experienced a variety of different appearance concerns among patients, even though few of them was working in appearance-related speciality units. The findings thus showed how appearance was experienced to be a more general aspect affecting the patients in nursing situations regardless of diagnosis or the ailment requiring care, which can be recognised from previous research [8]. Appearance issues in nursing situations are, according to the present study, strongly related to situations when the patients’ body is under scrutiny; intimate nursing situations as well as social situations within the nursing context. The participants’ experiences thus clearly showed the importance and impact of appearance issues in nursing situations in general and their link to the health of the patients. Previous research has also shown that appearance issues are important because of their relationship to health, particularly negative health consequences [1, 8, 14, 16]. The psychosocial impacts of appearance concerns among patients across the lifespan add to this importance and have frequently being reported by health care staff [8, 10]. The importance of appearance issues can be exemplified by previous research showing that approximately 36% of cancer patients experience appearance problems [27], weight concerns are common among pregnant women [28] and appearance issues are important in the care of patients with dementia [29]. In the present study the participants’ experience of appearance issues were also strongly related to the relationship between appearance concerns and negative health consequences, that is a pathogenic perspective [30]. Knowledge, awareness, and skills for prevention of patients’ appearance concerns from a pathogenic perspective are thus needed among nursing staff in general.

### Patients’ vulnerability

The findings showed that patient’s appearance-related vulnerability in nursing situations was related to gender, age, socioeconomic disadvantage and overweight. The participants’ experience of gender difference, with women being more vulnerable, can be related to a well-documented gender difference, with appearance ideals for women being more detailed, women being more susceptible to messages about appearance ideals and more often dissatisfied with appearance [31–33]. The age perspective, with young patients being experienced as more vulnerable than older, can be related to research showing that adolescents are especially susceptible to messages about appearance ideals in general and through media, with an associated risk for health-compromising behaviours and consequences [4, 33–36]. Research has shown a need for health care providers to promote positive body image from a more holistic perspective among patients in general [9]. A complementary salutogenic perspective for health promotion, focusing on resources for health, is characterised by a holistic perspective [30], and can thus facilitate promotion of positive body image in nursing situations. Positive body image is related to higher self-esteem, less depression and healthier dieting among women as well as men [9] and is therefore motivated in appearance-related recognition and support in nursing situations.

The participants described overweight patients as being especially vulnerable regarding appearance issues in nursing situations. Research has identified 46% of Swedish adults as being either overweight or obese [37] indicating that a large proportion of the population can be vulnerable regarding appearance issues as patients. Overweight and weight loss efforts are complex phenomena related for instance to gender and socioeconomic position. Inequalities in health are thus likely to be preserved or to increase because individuals with poor socioeconomic position are more likely to remain overweight, with the associated health risks [37]. Overweight being the opposite to the dominant thin and muscular ideal in mainstream Western culture [31, 36] also means a risk for discrimination. Weight discrimination leads to poorer mental health outcomes but also to an increased risk for obesity [38], with associated negative health consequences and inequalities. The many health risks of overweight should motivate initiatives for prevention and support in general, especially among children. The findings of this study showed, however, how initiatives for prevention of overweight among children within the school health system can also have an unintentional counter effect, emphasising the importance of nursing staff continuously reflecting on initiatives from an ethical perspective. Several moral concerns have been identified with a behaviour-change approach, and an empowerment approach is instead preferred for the prevention of victim blaming, stigmatisation and increased inequalities in health [39].

Stigmatisation and inequalities in health can also be related to the vulnerability of socioeconomic disadvantaged patients. “Health inequities arise from the societal conditions in which people are born, grow, live, work and age, referred to as social determinants of health” [40]*.* Health inequalities thus represent a complex public health problem in need of comprehensive initiatives on a societal level [40, 41], but also initiatives for empowerment on an individual level [42]. An empowerment approach for health is thus also suitable for preventing stigmatisation of the socioeconomic disadvantaged and vulnerable patients in nursing situations, associated with inequalities in health. The findings of the present study showed however how the vulnerability of patients is instead related to the opposite; that is patients with a subordinated role compared to the expert role of nursing staff in general. Research has shown that roles and relations between patients and health care providers can contribute to health disparities through varying quality of health care [17]. Major, Berry Mendes & Dovidio [17] have also shown that these intergroup processes can lead to health disparities through varying exposure to and experiences of chronic and acute stress as well as different health behaviours among patients from different social groups [17]. There is thus an opportunity for prevention of stigmatisation of socioeconomically disadvantaged patients, with an associated opportunity for prevention of inequalities in health and healthcare through an empowerment approach in nursing practice in general, including balancing of power between the roles of patients and nursing staff. Strategies for equity in health, with a focus on especially vulnerable groups are also warranted from an ethical perspective [43].

Appearance issues in nursing situations are a complex and challenging phenomenon for nursing staff in general. This study found that the complexity surrounding appearance issues was related to the participants’ experience of challenges in identifying, addressing, and supporting patients regarding appearance issues. Previous research confirms appearance issues as challenging for health care professionals and found a need for resources for improvement of appearance-related care [8, 10, 11, 27–29, 44]. Hospital settings are, according to the findings of the present study, equipped with extra resources for coping with appearance issues through the availability of a variety of professions, but previous research shows a need for clarification regarding different roles and access to referral [28]. Staff in appearance-related professions have been identified as more confident regarding patients’ appearance issues [8] and the findings confirm the need for knowledge, awareness, and skills among nursing staff outside appearance-related speciality units within the health care system.

### Values and priorities within the healthcare system

The participants’ experiences indicated contextual challenges within the Swedish tax-funded health care system, which was associated with limited resources and the prioritisation of objective physical functionality problems over subjective psychological concerns. Contextual factors in the health care sector have previously been identified as contributing to social inequalities [18] and are thus also important for reducing social inequalities in health [40]. The findings of the present study showed that prioritisation could be related to the orientation of the care setting but also to the values of nursing staff. The participants described staff looking down on ailments considered self-inflicted, for instance gastric bypass for prevention of overweight and plastic surgery to address dissatisfaction with appearance. Previous research confirms the existence of negative attitudes towards appearance issues among health care staff [44] indicating a need for value-based appearance-related awareness and development among nursing staff.

### Appearance-related first impression inferences

First impression inferences by health care staff, based on the facial appearance of patients, can also bias caring inclination in health care situations [20]. The risk of first impression inferences can be related to the participants’ descriptions of different strategies for professionalism with associated equality and equivalence of care. Some of the participants thought that all patients are treated equally because professional nursing staff automatically disregard patients’ appearance in nursing situations. Others believed however that disregarding patients’ appearance was impossible and therefore they emphasised the importance of awareness and continuous reflection concerning appearance-related first impression inferences. Previous research highlights the importance of awareness-raising among health care providers [17, 19, 20] for the promotion of equality and equivalence of care [14, 16, 17]. Experienced nurses are generally less biased by these inferences and react more to additional information about a patient than novice nurses [20], which can be related to the participants’ thoughts about professional experience as a resource for appearance-related awareness and support. There is thus a need for initiatives focusing on awareness of first impression inferences among prospective and novice nurses.

### Resources for appearance-related recognition and support

The participants, as well as previous research, highlighted the importance of awareness and recognition of appearance issues in nursing situations when needed [8, 27], but with sensitivity regarding the complexity of the phenomenon and the varying needs of the patients. The participants continuously referred to the strength of a holistic, person-centred nursing approach, which gives the opportunity for person-adjusted appearance-related awareness and support, without judgement and stigmatisation based on preconceptions or prejudices. A need for additional holistic approaches in care, including psychosocial resources for appearance-related issues, has been identified [8, 10] and holistic nursing as well as partnership are central components of person-centred care [45]. Person-centredness with a focus on the person/nurse meeting includes a focus on the social environment, personalisation, shared decision-making, communication, and empowerment [45] and can thus be related to the previously recommended empowerment approach [39]. An empowerment approach can thus support the implementation of appearance-related, person-centred care with a focus on the person/nurse interaction. The findings showed that experience of nursing practice could be a resource for delivering person-centred care. Important prerequisites for person-centred nursing are professional competence and self-awareness among health care professionals [46]. The participants, however, also highlighted that nursing experience could be an impediment to person-centredness, if the experience resulted in a habitual effect and more routine-based nursing practice. A habitual nursing practice is the opposite of the collective reflective learning process for professional competence requested by the participants, indicating that the risk for experience being an impediment can be reduced through systematic implementation of professional reflection forums for nursing staff. The need for awareness raising and knowledge development regarding appearance issues has been identified [8, 11, 44] as well as the benefit with reflective practice in health care [47] and especially collective learning processes for quality improvement of care [48]. Time is an important resource for participating in collective learning processes and the participants also emphasised the previously identified need for time allocation regarding appearance-related awareness and support [8, 28].

### Strengths and limitations

The methodological considerations are presented with focus on trustworthiness in qualitative research in general [25] and for conducting thematic analysis [24].

The use of participants who were also nursing students at a university can be considered as both a weakness and a strength of this study. It is a weakness regarding transferability since we do not know if there is a link between the participants being in education and the findings. It can however also be considered a strength in relation to the variety of experiences from different nursing contexts, as well as professional experiences being represented among the participants. Experience from varying nursing settings is considered especially important due to the exploratory character of the study. The participants’ differences in age and gender also strengthened the heterogeneity and transferability of this work, but with a limitation due to the absence of participants from the oldest professional age group, 45–65 years. The proportion of male participants adds to the heterogeneity of the study but might be a limitation regarding transferability, since the percentage of men is larger than in Swedish nursing contexts in general. However, no gender differences were identified in the experiences of the participants. The variety in the findings can be considered a verification of the heterogeneity and experience from the varying nursing settings of the participants. It also indicates that the climate of the focus group interviews supported the participants in sharing their varying experiences of appearance issues in nursing situations, with associated strengthening of the trustworthiness of the study findings.

All researchers participated actively throughout the reflexive process of analysis, which is considered a strength concerning the credibility of the findings. A summary of the findings was also member-checked and verified through peer debriefing, which is also a strength regarding credibility. The dependability of the study is supported by the detailed presentation of the research process and confirmability is strengthened by presenting the description of the analysis process and the findings in combination with citations from the focus group interviews.

## Conclusions

Patients’ appearance issues have an impact on nursing situations in general and can be related to the health of the patients. Some groups of patients are identified as more vulnerable than others, which is associated with health care inequalities and health disparities. Value-based strategies, together with knowledge, awareness, and skills among nursing staff, are thus needed for the prevention of appearance-related health care inequalities in nursing situations. Strategies for improvement can be realised through the educational system for nursing staff, but mainly using collective reflective learning forums in different workplaces. An empowerment approach is identified as a useful framework for the implementation of holistic person-centred care, functioning as a resource for appearance-related awareness and support in various nursing situations. However, more research is needed for further exploration of the complex and challenging phenomenon of appearance issues in nursing situations. Knowledge development related to successful person-centred strategies for appearance-related awareness and support is important and especially strategies with a salutogenic perspective.

## Data Availability

The datasets generated and analysed during the current study are not publicly available due to the confidentiality agreement with the participants. The data is however available from the corresponding author on reasonable request.
